# Fluid Dynamic Assessment of Hypersonic and Guillotine Vitrectomy Probes in Viscoelastic Vitreous Substitutes

**DOI:** 10.1167/tvst.9.6.9

**Published:** 2020-05-12

**Authors:** Alessandro Stocchino, Irene Nepita, Rodolfo Repetto, Andrea Dodero, Maila Castellano, Mariantonia Ferrara, Mario R. Romano

**Affiliations:** 1 Department of Civil, Chemical and Environmental Engineering, University of Genoa, Genoa, Italy; 2 Department of Chemistry and Industrial Chemistry, University of Genoa, Genoa, Italy; 3 Eye Center, Humanitas Gavazzeni-Castelli, Bergamo, Italy; 4 Department of Biomedical Sciences, Humanitas University, Milano, Italy

**Keywords:** fluidics of vitrectomy probes, flow measurements, hypersonic probes

## Abstract

**Purpose:**

To assess the fluidics of 23-gauge (G) large-port (L) and tear drop-port (TD) hypersonic vitrectomy probes (HVPs) compared with guillotine vitrectomy probes (GVPs) of various calibers (23G, 25G, and 27G) and geometries (single and double blades). Also, to identify the working parameters that provide the best balance between acceleration and flow rate, and, for HVPs, to measure temperature variations in the fluid.

**Methods:**

We used particle image velocimetry to measure flow fields in balanced salt solution and viscoelastic artificial vitreous. We analyzed acceleration, kinetic energy, and volumetric flux. The parameters considered were vacuum pressure, ultrasound stroke, and cut rate. Temperature measurements were taken using an infrared thermal camera.

**Results:**

The flow rate was significantly higher for HVPs than GVPs. With both probes, flow rate and acceleration increased with vacuum pressure. Flow rate depended weakly on the ultrasound stroke or cut rate. In HVPs, the acceleration peaked at a stroke of 30 µm, whereas in GVPs it peaked at a cutting rate of 4000 to 5000 cuts per minute (cpm). The HPV/TD combination generated higher flow rates and lower accelerations than did HPV/L. The increase in temperature was small.

**Conclusions:**

Under the present experimental setup and medium, HVPs offered better fluidics compared with GVPs in terms of flow and acceleration; however, the flow structure for HVPs is more complicated and unsteady. The HPV/TD combination produced larger flows than did the HPV/L combination and slightly smaller accelerations. HPVs generated a small temperature increase.

**Translational Relevance:**

In the tested artificial vitreous, HVPs were found to be more efficient in terms of generating lower acceleration for a given flow rate. The slight increase in temperature observed with HVPs is unlikely to be clinically significant.

## Introduction

Pars plana vitrectomy is the procedure of choice for surgical treatment of a wide range of vitreoretinal diseases. It is now well established that, in order to perform safe and effective surgery, it is important to minimize fluid accelerations while keeping sufficiently large values of the flow rate.^1^^,^^2^ This is because fluid acceleration and the consequent flow intermittency generate pressure variations, vitreoretinal tractions, and, potentially, iatrogenic breaks and/or removal of healthy tissue.^3^^,^^4^

Traditional guillotine vitrectomy probes (GVPs) have been optimized through progressive reductions in size and increases in cut rate.^5^ Recently, hypersonic vitrectomy systems have been introduced in which a handpiece, the hypersonic vitrectomy probe (HVP), produces high-frequency longitudinal vibrations with the aim of inducing vitreous liquefaction close to the tip.^6^^,^^7^ The potential advantages of HVPs are related to probe design and mechanism of action. With regard to the former, in a GVP the inner needle moves inside the outer one, determining the cyclic port opening and closing, whereas in the HVP there is a single needle and the port is always open. As a consequence, HVPs have a larger inner diameter than GVPs of the same nominal size. According to Poiseuille's law, the volumetric flux increases with the fourth power of the radius (the resistance to flow is proportional to the inverse of the fourth power of the radius). Thus, when using HVPs, one should expect significantly lower flow resistances, thus allowing the user to set the infusion pressure to lower values in order to keep the same flow rate.^8^ Moreover, HVPs have no limitations associated with the duty cycle, as the port is always open.^9^

Regarding the mechanism of action, the HVP is an ultrasonically, not pneumatically, driven probe, and rather than cutting the vitreous it is intended to cause its liquefaction. GVPs generate flow fluctuations that are inherently associated with the cutting process.^1^^,^^4^^,^^10^ Because there is no cutting with HVPs, fewer fluctuations are expected to occur. The cut rate of GVPs has been progressively increased with the aim of reducing the viscosity of the aspirated fluid^11^^,^^12^; however, the maximum cut rate is mechanically limited by the duty cycle and speed of the GVP blade.^13^^–^^15^ Moreover, the fragmented vitreous has complicated viscoelastic properties, and recent data have demonstrated that increasing the cut rate does not effectively reduce its viscosity.^16^ Unlike GVPs, HVPs are designed to produce vitreous liquefaction close to the tip.^7^

Based on these findings, HVPs offer several benefits in terms of fluidics. Recently, Stanga et al.^7^ and Rizzo et al.^17^ reported high flow rates of water, balanced salt solution (BSS), and vitreous using various HVPs and an ultrasound-based vitrector prototype, respectively.

On the other hand, it has been hypothesized that the use of HVPs might be associated with significant heat production, which can lead to temperature increases in the vitreous chamber, potentially causing thermal damage to the surrounding tissues.^18^

The aim of our study was to reliably measure the fluidics of 23-gauge (G) HVPs in the two available port configurations and compare those findings with GVPs under various vitrectomy settings. All of the experiments were performed in both BSS and artificial vitreous (AV) in order to simulate both liquefied and healthy vitreous conditions. Finally, in order to assess whether use of the HVP induces significant temperature increases, we took temperature measurements over time during operation of the HVP.

## Methods

### Experimental Setup

The experimental setup was similar to the one used in a previous work by the same research group.^1^ The measuring chamber consisted of a cubical reservoir (3 × 3 × 3 cm), with transparent Perspex walls; the reservoir was open on the top and filled with the working fluids. We performed experiments with various vitrectomy probes connected to the Stellaris Elite system (Bausch + Lomb, St. Louis, MO, USA). In particular, we tested four GVPs: 23SB (23G, single blade), 25SB (25G, single blade), 25BB (25G, double blade), and 27BB (27G, double blade). We also tested the Vitesse (VIT) 23G HVP (Bausch + Lomb) in two existing configurations of the port: large (L), 225 µm, and tear-drop (TD), 255 µm. The vitrectomy handpieces were kept in vertical position by a specifically designed holder and entered the measuring chamber from the top.

### Working Fluids

We used three different working fluids: BSS and two AV solutions, which we labeled S1 and S2, prepared as a solution of hyaluronic acid in deionized water based on the recipe proposed by Kummer et al.^19^ However, because the transparency of the fluid is a strict requirement for the optical measurements of the flow, we did not add the agar powder as suggested by these authors.

The rheological properties of each AV solution were tested with the Physica MCR 301 rotational rheometer (Anton Paar, Graz, Austria). In order to evaluate the complex modulus of the fluid, we performed oscillatory tests over a range of frequencies from 1 to 30 Hz with a shear strain of 0.05%, within the linear viscoelastic regime determined with preliminary amplitude sweep tests. The real part of it, *G*′, is a measure of the elastic response of the fluid, whereas the imaginary part, *G*′′, is a measure of the viscous response. Moreover, we tested fluid samples at a continuous rotational velocity and determined the stress–shear rate curve $\left(\tau -\dot{\gamma }\right)$, in the range 0 < $\dot{\gamma }$ < 1000 s^–1^, from which we obtained the value of the apparent viscosity $\mu =\tau /\dot{\gamma }$. All measurements were taken at 20°C.

**Figure 1.**
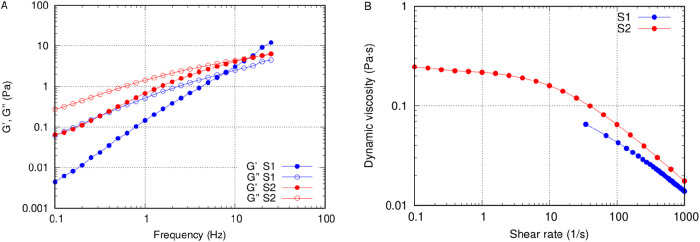
(**A**) Storage and loss moduli versus oscillation frequency (0.1–30 Hz); shear strain = 0.05%. (**B**) Apparent viscosity versus shear rate; temperature (T) = 20°C.

The results of rheological tests on the two AV solutions are shown in Figure 1. The rheological properties, in terms of complex nodulus (Fig. 1A), were reasonably close to those reported for porcine vitreous, even if the fluid has a smaller elastic modulus and a higher viscosity than the real vitreous.^20^^–^^22^ The solutions had shear thinning properties, with an apparent viscosity that decreased significantly as the shear rate increased (Fig. 1B). This is in good agreement with the rheological measurements that Silva et al.^23^ performed on the vitreous of rabbit eyes. Finally, solution S2 had a significantly higher complex modulus, in terms of both real and imaginary parts, at least at small frequencies.

### Particle Image Velocimetry and Post-Processing of the Experimental Data

The flow field generated by the vitrectomy probes was measured using particle image velocimetry (PIV), an optical, non-intrusive technique able to produce measurements of two-dimensional flow fields on planes illuminated by a laser sheet. We performed measurements on two vertical planes containing the probe: one parallel to the port aperture (front view) and one across it (lateral view). Moreover, in order to more accurately describe the flow structures for solution S2, two areas of interest were used for the acquisitions: (1) a large field the same size as the experimental box, with the aim of describing possible large-scale circulations; and (2) a small field, which was a zoomed area of about 11 × 13 mm around the cutter tip.

We determined whether the raw output of the PIV analysis—the velocity field ***u***(*x*, *y*, *t*)—was affected by systematic errors (bias and/or pixel locking effects) by computing the probability density function of the velocity magnitude. Moreover, we verified that the percentage of the outliers for each velocity fields never exceeded 3% of the total number of velocity vectors, which is an indication of the reliability of the measurements.

The measured velocity fields, ***u***(*x*, *y*, *t*), have been used to calculate fluid acceleration, ***a*** = ∂***u***/∂*t* + (***u*** · ∇)***u***, and kinetic energy per unit mass, *E_c_* = 1/2***u*** · ***u***.

In order to compare the behavior of vitrectomy probes employed in various conditions, we referred to synthetic quantities obtained by performing averages of the experimental measurements over time and space. For spatial averages, we adopted a circular averaging area, with a diameter of 6 mm, centered in correspondence with the port of the probe. Spatial averaging leads to time signals, which we analyzed by constructing power density spectra (PDS) of the kinetic energy per unit mass in order to identify the dominant frequencies of the flow. Time averaging, on the other hand, produces spatial maps that provide information about the mean flow characteristics. Finally, the result of averaging over time and space is a single quantity that is a synthetic measure of flow properties.

### Flow Rate Measurements

Flow rate measurements were taken tracking the free surface of the liquid in the measuring chamber over time. Automatic tracking was performed, and the digital images were analyzed with Motion Studio software (Integrated Design Tools, Pasadena, CA, USA). From time measurements of the free surface level, we extracted the surface speed by performing a linear regression of each signal. In particular, we adopted a linear regression based on the ordinary least-squares method. The error of the flow rate measurements was estimated computing the standard deviation of the coefficient representing the slope of the linear fitting multiplied by the chamber area, which turned out to be within the range of ±10^−2^ to 10^−3^ ml/min. Thus, the error was two or three orders of magnitude smaller than the measured flux. From knowledge of the vertical speed of the surface and of the cross-sectional area of the chamber we computed the flow rate.

### Temperature Measurements

We also performed temperature measurements for the HVPs. A Perspex floating panel was placed on the free surface of the experimental chamber in order to decrease thermal dispersion. We used a FLIR i7 infrared thermal camera (FLIR Systems, Inc., Wilsonville, OR, USA), which reconstructs a temperature map on the measuring plane based on measurement of the infrared radiation emitted by the fluid. Temperature measurements were taken only in BSS (as its thermal properties are very similar to those of AV) at zero vacuum pressure so that the amount of fluid in the domain remained constant over time. The temperature measurements obtained by the FLIR camera have been preliminarily validated against air and BSS temperature measurements performed with a standard thermometer with a resolution of 0.1°C. At the beginning of the test, the fluid was at room temperature (around 21.5°C), and the HVP was then operated continuously at a fixed stroke (the amplitude of the probe elongation) for 5 minutes, during which temperature was measured once every minute.

### Description of the Experiments

We performed different series of experiments varying the aspiration pressure and the cutting frequency (GVPs) or ultrasound stroke (HVPs). Both probes were tested with different working fluids. The Table provides a summary of all of the experiments, divided into three separate series depending on the working fluid (BSS, S1, or S2). It is worth noting that in the case of BSS we acquired only one view, as previous works suggested that, in this case, the flow has axial symmetry.^1^^,^^2^ Some experiments were repeated in order to verify the repeatability of the measurements.

**Table.** Parameters Characterizing the Experiments

Working		Pressure	Cuts Per		Field of	
Fluid	Cutter	(mm Hg)	Minute/Stroke	View	View	Measurements		
BSS	23SB	200–600	2000–7500	Front	Large field	Flow rate		PIV
	25SB	200–600	2000–7500	Front	Large field	Flow rate		PIV
	27BB	200–600	2000–7500	Front	Large field	Flow rate		PIV
	VIT-TD	300–600	5–60	Front	Large field	Flow rate	Temperature	PIV
	VIT-L	300–600	5–60	Front	Large field	Flow rate	Temperature	PIV
AV (S1)	23SB	200–600	2000–7500	Front/lateral	Large field	Flow rate		PIV
	VIT-TD	200–600	5–60	Front/lateral	Large field	Flow rate		PIV
	VIT-L	100–600	5–60	Front/lateral	Large field	Flow rate		PIV
AV (S2)	23SB	200–600	2000–7500	Front/lateral	Large/small field	Flow rate		PIV
	25SB	200–600	2000–7500	Front/lateral	Large/small field	Flow rate		PIV
	25BB	200–600	2000–7500	Front/lateral	Large/small field	Flow rate		PIV
	27BB	200–600	2000–7500	Front/lateral	Large/small field	Flow rate		PIV
	VIT-TD	100–600	5–60	Front/lateral	Large/small field	Flow rate		PIV
	VIT-L	100–600	5–60	Front/lateral	Large/small field	Flow rate		PIV

## Results

### Two-Dimensional Flow Fields

**Figure 2.**
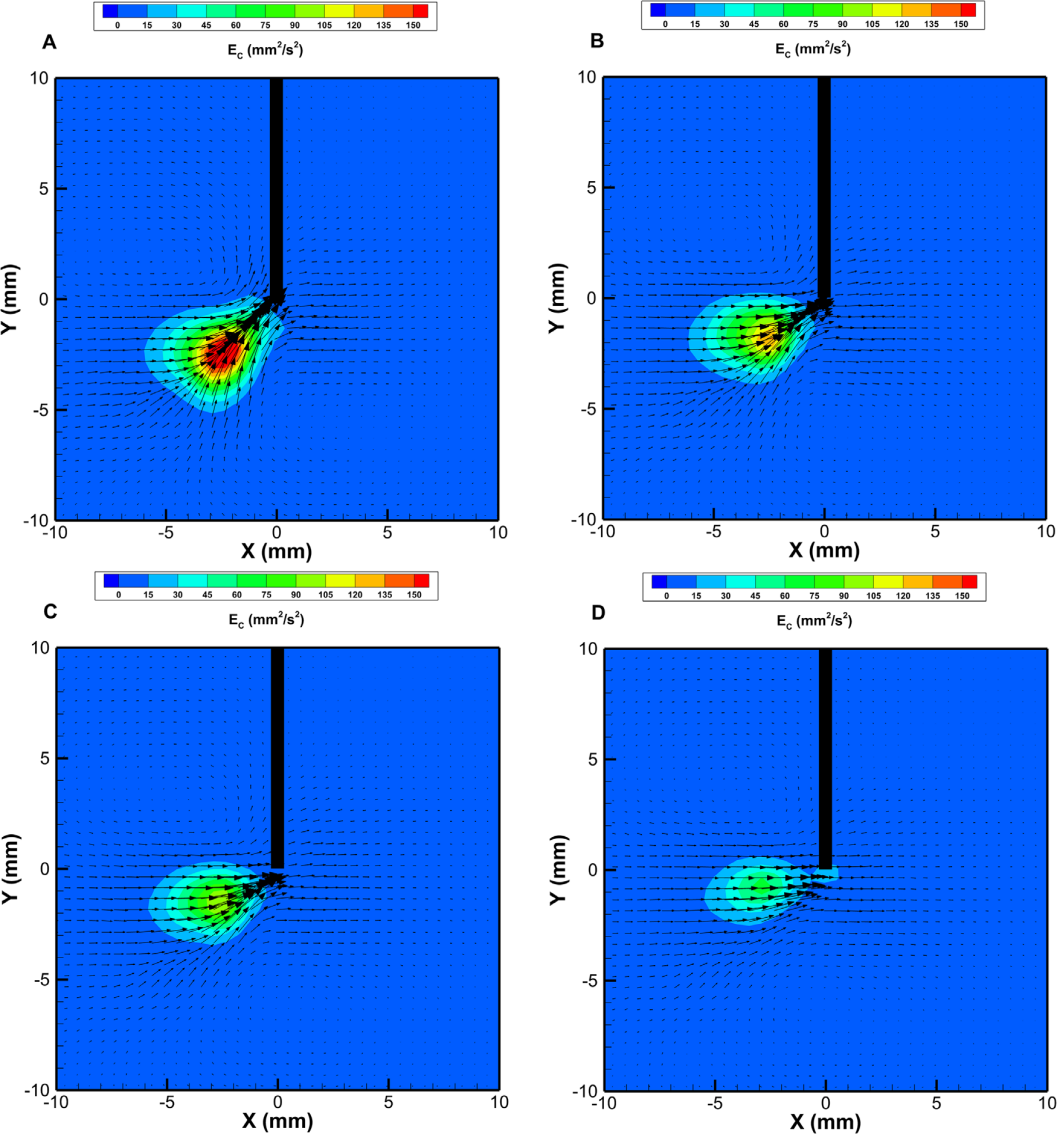
Time-averaged maps of the kinetic energy per unit mass and velocity vectors for the guillotine cutter. Lateral view with the cutter port on the negative side of the *x*-axis; 23G cutter with pressure set at 600 mm Hg. The plots correspond to the various cutting frequencies (*f*): (**A**) *f* = 2000 cpm; (**B**) *f* = 4000 cpm; (**C**) *f* = 5000 cpm; (**D**) *f* = 7500 cpm.

In agreement with our findings in a previous study,^1^ the velocity distributions in BSS for both GVPs and HPVs were axisymmetric with respect to the needle and decayed monotonically with distance. The flow fields in AV differed significantly from those in BSS. Some examples obtained with GVPs in AV are shown in Figure 2. The flow fields in AV were highly asymmetric, and, on a lateral plane, a confinement region developed, inside of which fluid velocity was large and directed toward the port and outside of which was very small. This happened with all of the GVPs, regardless of the gauge and operating conditions. The confinement region had approximately a conical shape, and its axis formed an obtuse angle with the cutter axis, which decreased as the cutting frequency increased (Fig. 2). Similar results were found by Romano et al.^1^ We note that the instantaneous velocity fields were qualitatively similar to the time-averaged ones, as relatively small fluctuations in the time scale of the cutting cycle were superimposed onto the averaged flow (not shown).

Information about the time dependency of the flow can be inferred by inspecting the PDS reported in Figure 4A. Clear peaks were found corresponding to the cutting frequency, both its super- and sub-harmonics. For example, referring to the curve corresponding to a cutting frequency of 2000 cuts per minute (cpm), or 33.3 Hz, we found peaks at 16.7 Hz, 33.3 Hz, and 66.6 Hz. In this case, a peak also existed at 50 Hz, which is a frequency unrelated to the cutting and was probably a natural frequency of oscillation of the viscoelastic fluid in the measuring chamber.^1^^,^^24^

**Figure 3.**
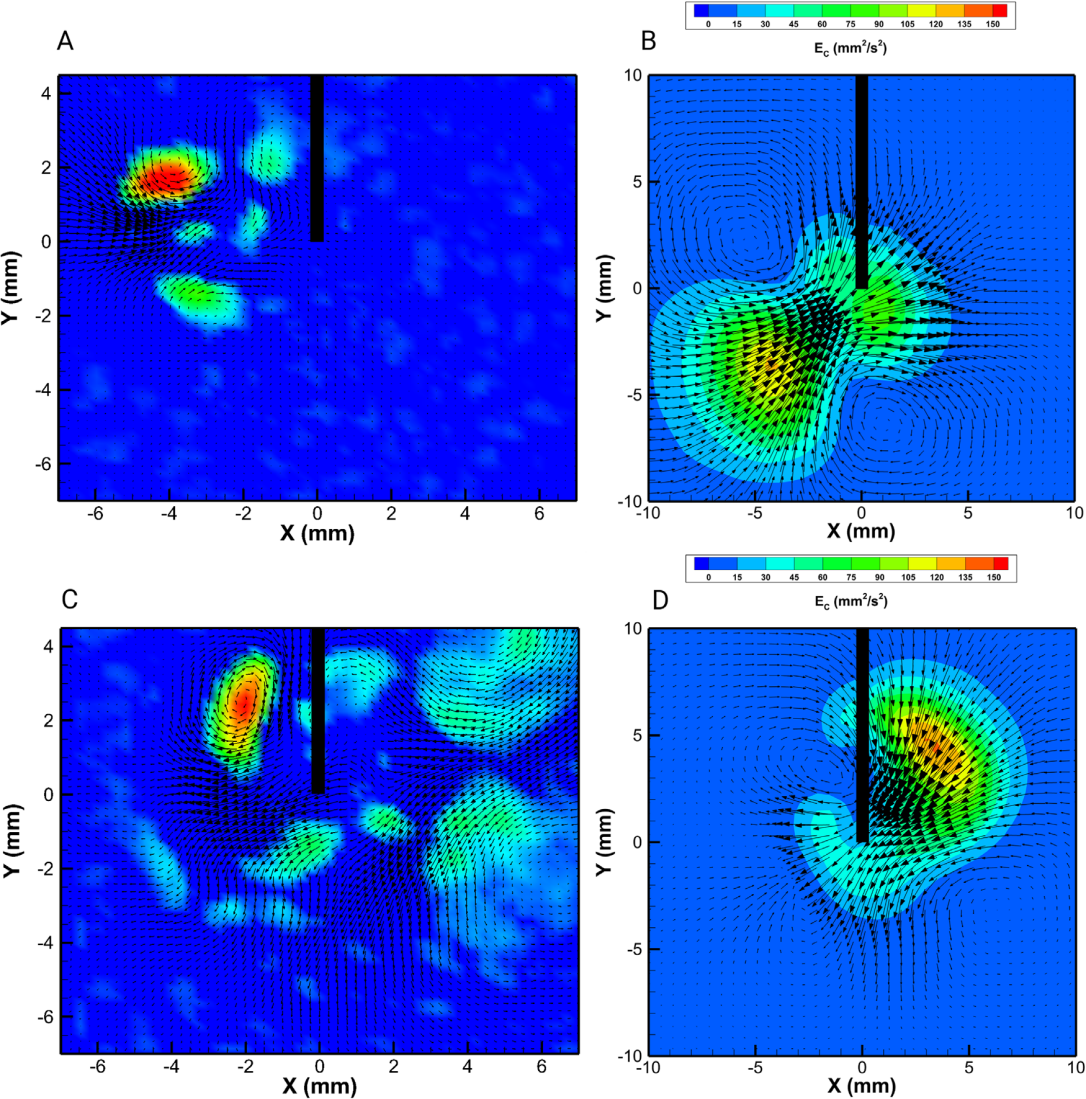
(**A**, **C**) Instantaneous and (**B**, **D**) time-averaged maps of the kinetic energy per unit mass and corresponding velocity vectors for the ultrasound VIT-L probe. Lateral view with the cutter port on the negative side of the *x*-axis; 23G cutter with pressure set at 600 mm Hg. The plots correspond to various stroke values: (**A**, **B**) 10 µm; (**C**, **D**) 30 µm.

**Figure 4.**
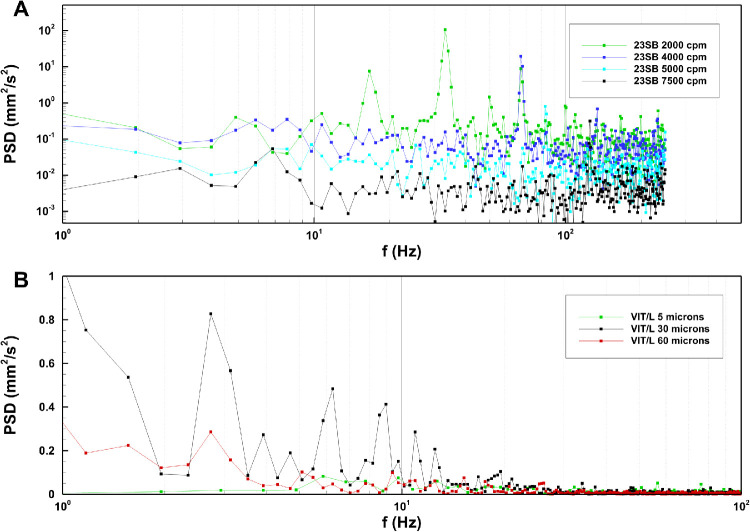
PDS of the kinetic energy per unit mass obtained for solution S1: (**A**) guillotine cutter 23SB; (**B**) ultrasound VIT-L probe. Note that the PDS obtained with S1 and S2 were qualitatively similar.

The flow field induced by HVPs was significantly different from that produced by GVPs, as shown in Figure 3. The instantaneous flow maps (Figs. 3A, 3C) were highly irregular, and, even when the pumping pressure was kept constant in the experiments, the flow was unsteady. Changes in time had frequencies much smaller than for the ultrasound probes, as shown in the PDS reported in Figure 4B. Flow unsteadiness can be appreciated in Supplementary Movies S1 to S4. Supplementary Movies S1 and S2 are the image acquisitions recorded for the VIT-L probe from frontal and lateral views, respectively, during an experiment performed with pressure equal to 600 mm Hg and stroke equal to 30 µm. Supplementary Movies S3 and S4 are the corresponding velocity vector fields. This behavior is likely due to the onset of flow instabilities, which is a typical occurrence in fluid mechanics. Of course, in the time-averaged flow fields the velocity was, on average, directed toward the port (Figs. 3B, 3D); however, the spatial structure of the time-averaged flow field varied significantly with the stroke, as can be seen by comparing Figures 3B and 3D.

Another peculiar finding for HVPs was the generation of fluid flow even in the absence of vacuum pressure, which was, therefore, entirely due to the oscillations of the ultrasound probe. The occurrence of steady flows produced by oscillating bodies in a fluid is a well-known phenomenon in fluid mechanics and is referred to as steady streaming, which occurs in both viscous^25^^,^^26^ and viscoelastic fluids.^27^ Cavitation has never been observed.

### Averaged Results

**Figure 5.**
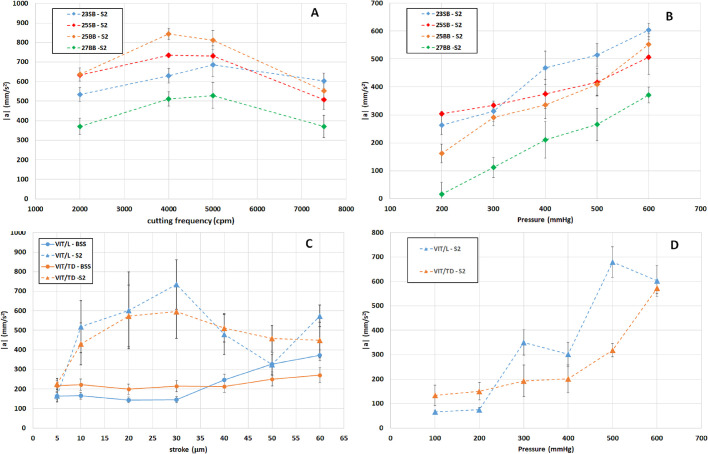
(**A**) Space and time averages of the magnitude of fluid acceleration as a function of cutting frequency for the guillotine cutter with solution S2, with fixed pressure set at 600 mm Hg. (**B**) Space and time averages of the magnitude of fluid acceleration as a function of pumping pressure for the guillotine cutter, with a fixed cutting frequency equal to 7500 cpm. (**C**) Space and time averages of the magnitude of fluid acceleration as a function of stroke for the ultrasound probe, with vacuum pressure set at 600 mm Hg. (**D**) Space and time averages of the magnitude of fluid acceleration as a function of pumping frequency for the ultrasound probe with solution S2, with a stroke of 20 µm.

We focused on two quantities: time- and space-averaged magnitude of fluid acceleration and volumetric flux (or flow rate). The values of the averaged acceleration displayed in the following are based on the PIV measurements performed in the small field and are averaged among the lateral and front view data.Figure 5 shows the average acceleration as a function of the cutting frequency for GVPs (Fig. 5A), of the stroke for HVPs (Fig. 5C), and of the pumping pressure for all probes (Figs. 5B, 5D). For GVPs, the acceleration peaked at a cutting frequency of 4000 to 5000 cpm (Fig. 5A). For HPVs, the acceleration grew slightly with the stroke in BSS experiments. On the other hand, with the AV, it reached a maximum in a range of strokes between 20 and 40 µm (Fig. 5C). Finally, for all probes, the acceleration grew monotonically with the pumping pressure (Figs. 5B, 5D).

**Figure 6.**
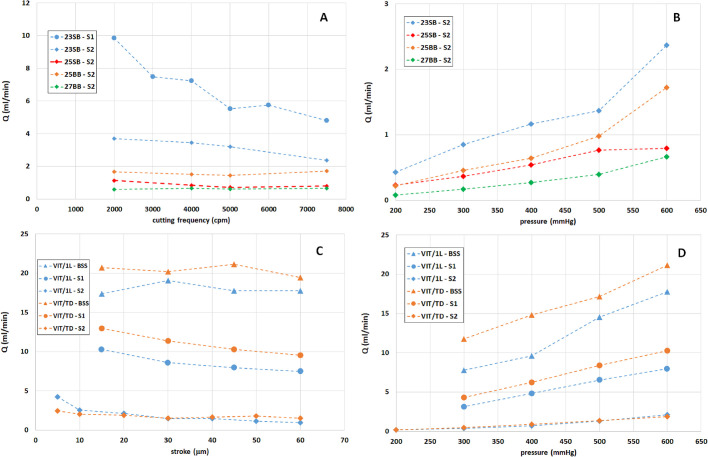
(**A**) Flow rate versus cutting rate for the guillotine cutter, with vacuum pressure set at 600 mm Hg. (**B**) Flow rate versus pumping pressure for the guillotine cutter at a cutting frequency of 7500 cpm. (**C**) Flow rate versus stroke for the ultrasound probe with vacuum pressure set at 600 mmHg. (**D**) Flow rate versus pumping pressure for the ultrasound probe with a stroke of 20 µm.

Figure 6 shows the results obtained measuring the flow rate, *Q*, as a function of cutting frequency for GVPs (Fig. 6A) and stroke for HVPs (Fig. 6C). Moreover, in panels B and D, we show the dependency of *Q* on pumping pressure for all probes. For GVPs, the flow rate was obviously higher the larger the needle; it decreased slightly with cutting frequency in the case of the 23G, whereas it was almost constant for the other gauges (Fig. 6A). For HVPs, the flux was approximately independent of stroke in BSS and slightly decreased with it in AVs. The TD port was always characterized by larger fluxes than the L port. For all of the probes, the flux increased as the pumping pressure grew (Figs. 6B, 6D).

### Temperature Measurements

**Figure 7.**
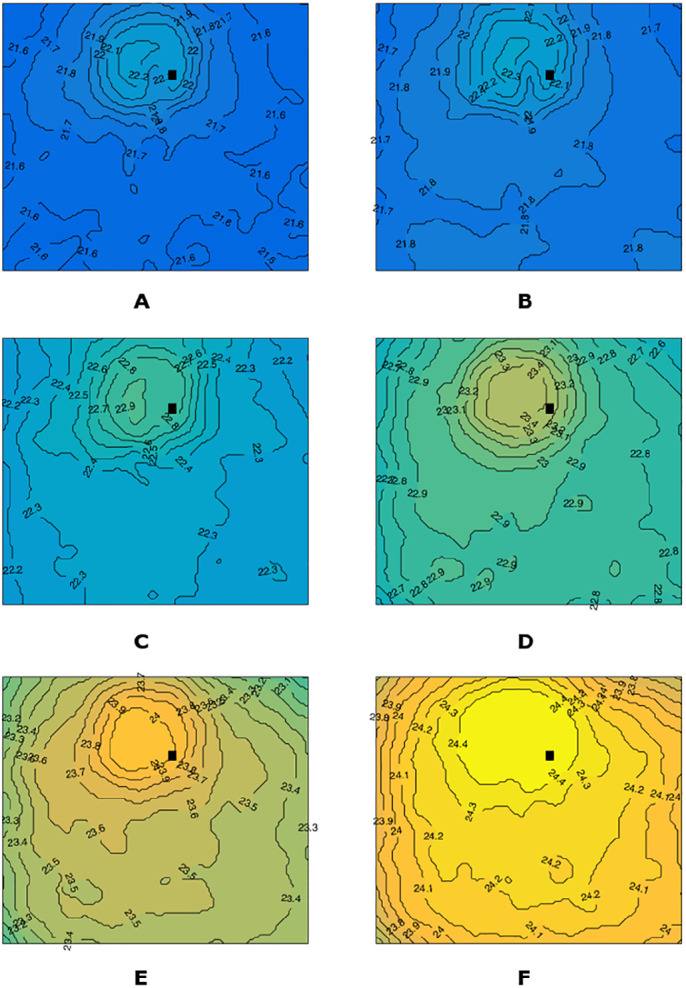
Temperature maps at different times *t* for the ultrasound VIT-L probe: (**A**) *t* = 0; (**B**) *t* = 1 minutes; (**C**) *t* = 2 minutes; (**D**) *t* = 3 minutes, (**E**) *t* = 4 minutes, (**F**) *t* = 5 minutes. Stroke set at 60 µm. The area depicted in the picture has a side of approximately 2 cm. The black square in the images indicates the approximate position of the probe tip. Temperature ranged from 21°C to 24.5°C.

**Figure 8.**
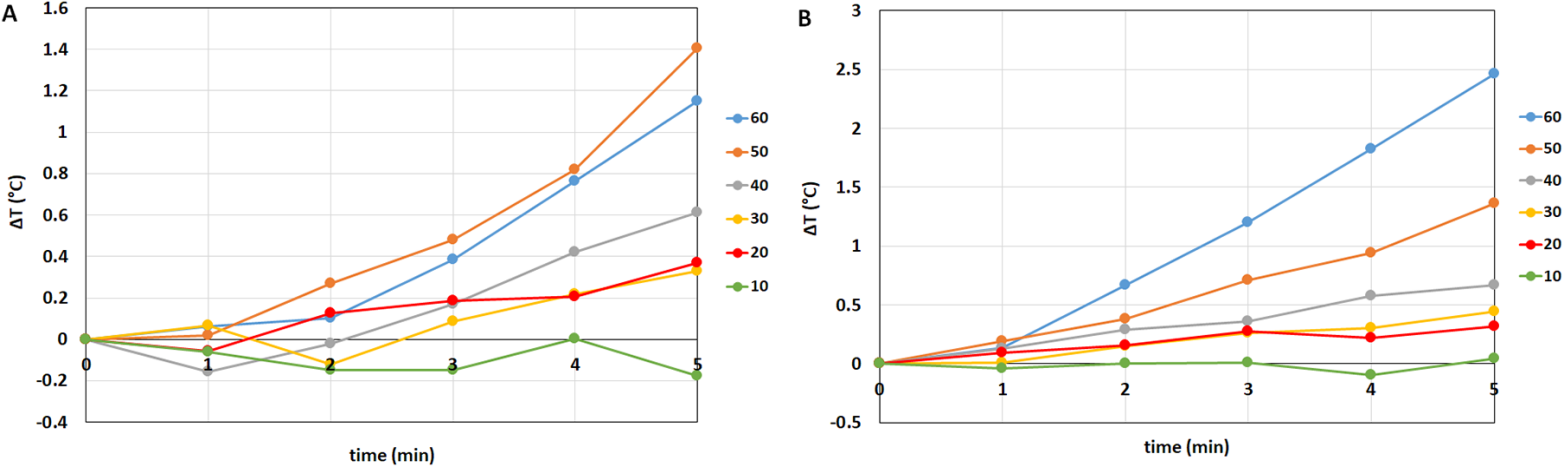
Temperature variation (∆*T*) over time for different strokes: Δ*T* = *T* – *T*_0_, where *T* is the actual temperature and *T*_0_ is the initial temperature. (**A**) VIT-L; (**B**) VIT-TD.

Temperature measurements were taken during use of the HVPs. A sequence of temperature maps at different times is shown in Figure 7. Because temperature gradients within the domain were quite small, the following text refers to the average temperature over the measuring plane. Temperature evolutions in time are shown in Figure 8, and we found similar results comparing VIT-L and VIT-TD. In particular, no significant temperature change was observed when the stroke was ≤30 µm, whereas temperature began to increase for higher strokes, reaching a maximum increase of about 2.5°C after 5 minutes with the VIT-TD operated at a stroke equal to 60 µm (Fig. 8). The temperature of the probe head, which we also monitored over time, never exceeded a temperature of 40°C.

## Discussion

Ultrasound-based vitrectomy systems have been recently introduced as promising alternatives to the traditional guillotine-based cutters (GVPs).^7^^,^^28^ HVPs possibly have several advantages over GVPs,^7^ such as the design of the probe that includes a single needle and a port that is always open, as well as the fact that vitreous liquefaction is likely to be induced, thus facilitating vitreous aspiration. These effects are thought to have a favorable influence in terms of steadiness of the flow, which is widely recognized as a crucial point for safe and effective surgery. So far, however, our knowledge about the effect of HVPs on vitreous properties and characteristics of the flow generated in the vitreous chamber is very limited.^7^

In the present study, we investigated experimentally the fluidics of two HPVs (VIT-L and VIT-TD) and compared them with four GVPs (23SB, 25SB, 25BB, and 27BB). We performed the experiments in a cubical measuring chamber, using both BSS and AV as working fluids, in order to simulate both liquefied and healthy vitreous conditions.

The choice of an open cubical domain implies that pressure values generated in the fluid will differ from those in the eye, which is a pressurized organ. However, the intraocular pressure is small compared to the aspiration pressure imposed on the vitrectomy device, which means that the flow rate would not significantly change if a pressurized chamber was adopted. Moreover, pressure variations associated with fluid flow are correctly reproduced in our model, as fluid motion is produced by pressure gradients so there is gauge freedom in the pressure.

We also note that, during vitrectomy, fluid is pumped out of the vitreous chamber by the vitrectomy probe and is replaced with fluid entering from an infusion line; therefore, the overall circulation in the vitreous chamber is likely to differ from what we measured, as we do not account for infusion. However, we are mostly interested in fluid motion in the vicinity of the vitrectomy probe tip, which is not likely to be significantly affected by the overall circulation in the domain.

The use of an artificial rather than a real vitreous can obviously be seen as a limitation of the present approach, as measurements on a real vitreous would more closely mimic actual surgical conditions. However, the use of AV also offers significant benefits, in that the experiments are reproducible and the rheological properties of the fluid can be accurately measured and controlled. This is not the case with real vitreous, as it is well known that the properties of the vitreous body can vary significantly among individuals, with both age and location in the vitreous cavity. Moreover, measuring fluid flow in the vitreous cavity is very challenging, and the approaches proposed so far do not allow one to obtain temporal or spatial resolutions even nearly comparable to those for in vitro experiments.^26^ We, therefore, believe that our approach complements similar studies performed on real vitreous humor, which better reproduce surgical conditions but cannot provide a deep description of the fluid mechanics events occurring close to the tip of the vitrectomy probe and the results of which are difficult to generalize.

For tests in BSS, the flow field was invariably found to be axisymmetric. The situation was significantly more complicated in the case of AV. In particular, for all tested GVPs the flow in the AV developed a confinement region in front of the tip port (Fig. 2). This result is consistent with previous observations in both AV^1^ and egg albumen.^2^^,^^10^ The possible generation of confinement regions in viscoelastic and anisotropic viscous fluids is a known phenomenon in fluid mechanics and has been investigated in various contexts, such as the flow through an abrupt narrowing^30^^–^^32^ and into an orifice,^33^ which, similarly to the flow produced by a vitrectomy probe, is accelerated. From the surgical point of view, the generation of a confinement region characterized by large velocities and its extension and orientation are relevant whenever the cutter is used in the proximity of the retina, because, in that case, the orientation of the cutter port has a significant influence on the stresses on the retina. The flow generated by GVPs was also characterized by time fluctuations related to cutting frequency. In addition, in viscoelastic fluids, we also found one additional frequency with a high energy content that is likely to be related to a natural frequency of oscillation of the fluid in the domain.^1^^,^^4^^,^^24^

For HVPs, the flow was highly unsteady and irregular in space. Unsteadiness in flows generated by steady mechanisms (such as a steady pumping pressure) is very common in fluid mechanics and is due to the onset of instabilities. This typically happens when the Reynolds number (*Re* = *U* · *L*/*v*, where *U* is a typical velocity, *L* is a characteristic length scale, and *v* is the kinematic viscosity of the fluid), a measure of the relative importance of inertial over viscous forces, is large enough. In the case of flows induced by vitreous cutters, *Re* is at most of order 10 (*Re* computed with *L* equal to the probe diameter and with the viscosity estimated at large shear rates), which is not likely to result in the onset of hydrodynamic instabilities. However, in viscoelastic fluids, elastic instabilities are also known to possibly arise, even in the limit *Re* → 0^34^ and this might explain the phenomena observed in our tests. A deeper understanding of the mechanisms underlying our observations is complicated by a lack of knowledge regarding the effects that HVPs have locally on fluid properties.

We observed the generation of a steady flow (steady streaming), in the absence of an applied vacuum, that can be attributed to the periodic vibrations of the probe.^24^

Finally, we never detected the occurrence of cavitation, even at high ultrasound power.^17^ This is an important finding, as cavitation is highly undesirable in vitrectomy because it could disrupt the retinal tissue.

In order to characterize the efficiency of HVPs compared with GVPs, we focused on two synthetic quantities: flow rate and average fluid acceleration.^1^^,^^2^^,^^4^ With regard to GVPs, our results are consistent with previous studies.^1^^,^^4^^,^^7^^,^^10^^,^^16^ On the other hand, only two studies considered the flow rate generated by ultrasound-based vitrectomy systems, and none of them measured fluid acceleration or any other physical quantity related to the fluidics of HVPs.^7^^,^^17^ Thus, this is, to our knowledge, the first study to provide a comprehensive assessment of the fluidics of hypersonic vitrectomy systems.

We found that flow rate and acceleration had an approximately linear dependence on the pumping pressure and that flow rate was almost independent of the stroke for both BSS and AV. This last result is in agreement with the work by Stanga et al.^7^ and Rizzo et al.^17^ for BSS. On the other hand, on porcine vitreous, the authors found that the flow rate increased with increasing ultrasound power, although Rizzo et al.^17^ observed that this increase was small and not invariably present. This difference with respect to our findings is possibly related to the use of a different medium. Acceleration, on the other hand, changed significantly with stroke and peaked for stroke values ranging from 20 to 40 µm.

**Figure 9.**
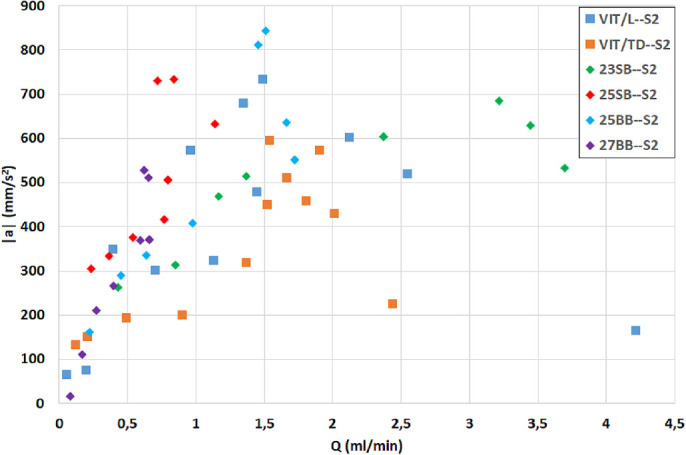
Diagram showing, for each experiment, flow rate versus time- and space-averaged magnitude of fluid acceleration.

In a seminal paper in the field, Rossi et al.^29^ proposed estimating the safety and efficiency of vitrectomy probes using a scatterplot in the plane (*Q*, |***a***|) (i.e., flow rate and acceleration) for a given set of controlling parameters. The rationale behind this approach is that a vitrectomy system is efficient if it allows the surgeon to perform surgery in a short time (which implies large flow rates), and it is safe if it does not generate large stresses on the retina (which implies small fluid accelerations). Accordingly, we plotted the results of our experiments in the (*Q*, |***a***|) diagram shown in Figure 9. Overall, the results suggest that HVPs offered better performance than the GVPs; for a given flux, accelerations were (on average) smaller for HVPs than for GVPs. Among the GVPs, the 25BB was found to perform better than the 25SB, as it produced higher flow rates with similar accelerations. This is consistent with previous studies comparing the fluidics of single-blade and double-blade cutters of the same size.^1^^,^^4^ Comparing the two HVPs we found that the VIT-TD offered better performance, as it produced larger flows regardless of the stroke. The superiority of the VIT-TD over the VIT-L has also been documented by Stanga et al.^28^

With regard to the 27BB GVP, under our experimental setup and medium we observed that no flow rate was produced at all when the fluid was very viscous and elastic (the corresponding points are not reported in Fig. 9). This could be related to the extremely reduced internal lumen of the needle and obviously depends on the properties of the AV considered.

Finally, we measured temperature variations in the fluid during HVP operation, which is an important factor because high temperatures can damage ocular tissues.^18^ In our experiments, we found a maximum temperature increase of 2.5°C after 5 minutes of continuous use of the HVP at a stroke of 60 µm. During the experiment, the temperature of the head of the HVP also increased, to a maximum of approximately 40°C. We note that, in terms of temperature variations in the fluid, our experimental setup did not accurately reproduce what happens during surgery, as we did not have an infusion line and the vacuum pressure was set to zero for temperature measurements, implying that there was no fluid exchange in the measuring chamber. During vitrectomy surgery, on the other hand, vitreous is pumped out of the eye and is replaced by a fluid that typically has a significantly lower temperature. This would drastically mitigate the tendency of the HVP to increase fluid temperature, which is already very small. For these reasons, we think that high temperatures in the vitreous chamber during vitrectomy with HVPs are not an issue.

## Conclusions

We assessed the fluid dynamic performance of vitrectomy probes in BSS and AV using a cubical measuring chamber. This study confirmed that flow rate and acceleration grow with aspiration pressure for all vitrectomy probes. Flow rate exhibited a weak dependence on cutting frequency and stroke for GVPs and HVPs, respectively, whereas acceleration peaked at 4000 to 5000 cpm for GVPs and between 20 and 40 µm for HVPs. Overall, the HVPs performed better than the GVPs, producing lower acceleration for a given flow rate. However, the HVPs produced an irregular and time-dependent flow, probably due to the onset of flow instabilities. Temperature elevation during surgery is very unlikely to be an issue.

## Supplementary Material

Supplement 1

Supplement 2

Supplement 3

Supplement 4
